# The Influence of Ca on Mechanical Properties of the Mg–Ca–Zn–RE–Zr Alloy for Orthopedic Applications

**DOI:** 10.3390/jfb16050170

**Published:** 2025-05-09

**Authors:** Mircea Cătălin Ivănescu, Corneliu Munteanu, Ramona Cimpoeșu, Bogdan Istrate, Fabian Cezar Lupu, Marcelin Benchea, Eusebiu Viorel Șindilar, Alexandru Vlasa, Ovidiu Stamatin, Georgeta Zegan

**Affiliations:** 1 Faculty of Dental Medicine, “Grigore T. Popa” University of Medicine and Pharmacy, 16 University Street, 700115 Iasi, Romania; mircea-catalin.ivanescu@umfiasi.ro (M.C.I.); ovidiu.stamatin@umfiasi.ro (O.S.); georgeta.zegan@umfiasi.ro (G.Z.); 2 Faculty of Mechanical Engineering, “Gheorghe Asachi” Technical University of Iasi, 43 Dimitrie Mangeron Blvd, 700050 Iasi, Romania; corneliu.munteanu@academic.tuiasi.ro (C.M.); bogdan.istrate@academic.tuiasi.ro (B.I.); marcelin.benchea@academic.tuiasi.ro (M.B.); 3 Faculty of Materials Science and Engineering, “Gheorghe Asachi” Technical University of Iasi, 41 Dimitrie Mangeron Blvd, 700050 Iasi, Romania; ramona.cimpoesu@academic.tuiasi.ro; 4 Surgery Unit, Clinics Department, Faculty of Veterinary Medicine, Iasi University of Life Sciences, Ion Ionescu de la Brad, 700490 Iasi, Romania; 5 Faculty of Dental Medicine, George Emil Palade University of Medicine, Pharmacy, Science, and Technology, 540139 Târgu-Mureș, Romania; alexandru.vlasa@umfst.ro

**Keywords:** calcium concentration, microstructure, scanning electron analysis, EDS, XRD

## Abstract

Background: This study examined how the concentration of calcium (Ca) influences the microstructure, mechanical characteristics, and tribological attributes of Mg–Ca–Zn–RE–Zr alloys for orthopedic medicine. Materials and methods: Experimental alloys with 0.1 and 0.5 wt% Ca were prepared in a controlled atmosphere induction furnace. The microstructure of the alloys was investigated by scanning electron microscopy, the chemical composition by X-ray fluorescence and energy-dispersive spectroscopy, the mechanical properties by indentation and scratching, and the corrosion resistance by linear and cyclic potentiometry. Results: The alloy with 0.1% Ca exhibited greater fluctuations in the coefficient of friction, while the sample with 0.5% Ca showed a higher susceptibility to cracking. Regarding corrosion resistance, both samples exhibited a generalized corrosion trend with similar corrosion currents. At lower Ca concentrations (0.1%), the refined microstructure of the alloys provided an elastic modulus closer to that of human bone, minimizing the risk of excessive local stress and promoting uniform load distribution at the bone-implant interface. Conclusion: The 0.5% Ca alloy offered superior tribological stability and better shock absorption, making it suitable for applications requiring long-term stability. The study highlighted the potential of both compositions based on the specific requirements of biodegradable orthopedic implants.

## 1. Introduction

The third generation of biomaterials, the biodegradable ones, represents a significant challenge, particularly in orthopedic systems. Magnesium (Mg) alloys, due to their low density, excellent biocompatibility, and biodegradability, have drawn significant interest as materials for resorbable implants [1]. However, limitations such as their rapid biodegradation and corrosion rate, as well as relatively low mechanical strength compared to other metals used for implants (stainless steel, titanium), have necessitated performance improvements through composition and microstructural optimization. Complex compositions have been explored by adding biocompatible elements that increase mechanical properties and degradation time and align it with the bone healing period [2,3].

The Mg–Ca–Zn–RE–Zr system represents a novel approach for the development of advanced magnesium alloys, owing to the synergistic interaction between the alloying elements, particularly Ca and Zn, and the standardized WE43 alloy, which has been extensively tested and studied, and has already received FDA approval for orthopedic applications under the commercial name Magnezix, manufactured by Syntellix (Syntellix AG, Hannover, Germany) [4]. The majority of previous research on Mg–Ca alloys has focused on Ca concentrations ranging between 0.4 and 10 wt.%, making it relevant to further explore the effects of lower concentrations.

According to [5], a comprehensive review of the literature indicated that Ca additions in Mg-based alloys within the 0.4–10 wt.% range contribute to grain refinement, thereby improving mechanical strength and reducing the corrosion rate through the formation of a protective Mg(OH)_2_ layer. However, increasing Ca content above 1 wt.% can lead to the excessive presence of the intermetallic Mg_2_Ca phase, which enhances the susceptibility to localized corrosion and material embrittlement, thereby limiting the biomedical applicability of such alloys.

Furthermore, reducing the Zn content while balancing the Ca concentration has led to the development of a high-strength, low-corrosion-rate alloy, Mg–0.45Zn–0.45Ca (wt.%) (ZX00), as demonstrated by Holweg et al. (2020) [6]. Although Zn-based alloying systems exhibit good mechanical strength and osteoinductive properties, excessive Zn release during degradation has been reported to induce toxicity at high concentrations [7].

Because it forms with Mg and Ca stable intermetallic phases like Ca_2_Mg_6_Zn_3_, zinc (Zn), at a concentration of about 1.5 weight percent, plays a significant role in strengthening solid solutions and reducing corrosion [2,3,8]. Up to 5 weight percent Zn can enhance the mechanical characteristics of magnesium alloys, especially by causing intermetallic precipitates to form [9,10]. Because Mg is an essential everyday element in the human body, this alloy system is perfect for orthopedic applications and also plays an important role in bone regeneration [11,12]. By creating intermetallic phases like Ca_2_Mg_6_Zn_3_, which stabilize the surface structure and slow the spread of corrosion, adding Zn to the Mg–Ca alloy greatly increases corrosion resistance [13]. In physiological settings, secondary phases such as Ca_2_Mg_6_Zn_3_ and Mg_2_Ca encourage the formation of continuous passive layers that improve corrosion resistance. Recent studies have shown that Ca concentration variations directly affect the microstructure by reducing grain size and stabilizing intermetallic phases. Zn is involved in bone formation by stimulating osteoblasts and inhibiting osteoclasts, while its deficiency can be a risk factor for poor calcification of the extracellular matrix. Zn is an essential trace element with a structural and functional role in the human body. Its function in bone remodeling and health is highlighted by the fact that changed zinc levels are linked to oxidative imbalances and biological reactions in malignancies or bone regeneration processes [14].

Rare earth (RE) elements like Yttrium (Y) and Gadolinium (Gd) enhance thermal stability and form intermetallic phases that limit corrosion propagation through galvanic mechanisms [15,16,17,18]. Meanwhile, the addition of zirconium (Zr) contributes to grain refinement, improving mechanical properties and fatigue behavior [19]. Adding Ca in variable concentrations (0.5–4 wt.%) brings significant benefits by increasing mechanical strength and further reducing the corrosion rate [16,19,20]. Ca concentrations up to approximately 1 wt.% in Mg-based alloys enhance biocompatibility, producing suitable materials for orthopedic implants [8,16]. Ca contributes to homogenizing the microstructure, resulting in improved corrosion behavior and increased fatigue resistance [21,22,23].

Heat treatments are helpful for regulating microstructure and enhancing these alloys’ mechanical and corrosion resistance [24]. Microstructures with finer secondary phases were produced by carefully regulating the heat treatment parameters (temperature and time). This resulted in the formation of more effective corrosion-resistant layers and a notable strengthening of the alloy because of the formation of nanometric precipitates [25,26].

The present study focuses on analyzing the electrochemical, mechanical, and microstructural behavior of the Mg–Ca–Zn–RE–Zr system with 1.5% Zn and varying Ca concentrations (0.1% and 0.5%). The primary objective is to evaluate the influence of Ca on microstructure, corrosion resistance, and fatigue behavior to develop an optimal material for biomedical applications. The study emphasizes the fundamental mechanisms governing protective layer formation, its stability, and interactions with physiological environments. The obtained results will contribute to a better understanding of the key parameters necessary for designing advanced Mg alloys for biodegradable implants.

## 2. Materials and Methods

### 2.1. Alloy Preparation Through Casting and Heat Treatment

**Figure 1:**
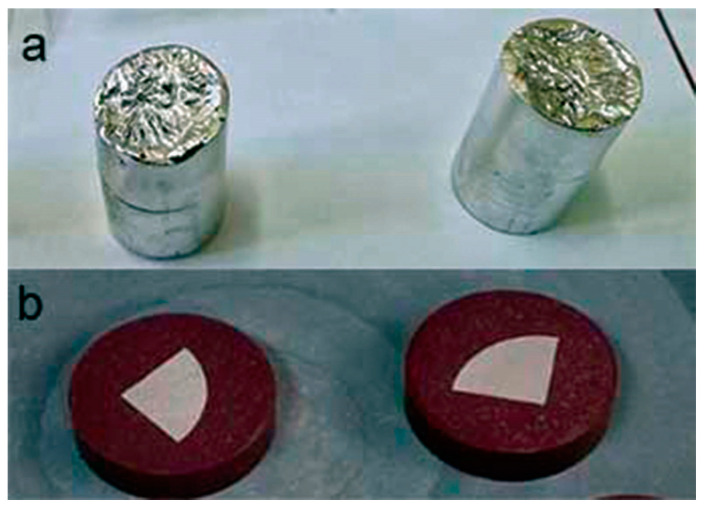
(**a**) Alloy ingots, (**b**) embedded alloys in resin prepared for analysis.

The Mg–XCa–1.5Zn–RE–Zr system (x = 0.1, 0.5%) was developed using the casting process in ceramic crucibles with an induction current system under an argon atmosphere to prevent alloy oxidation, employing the Rotocast casting machine. The alloy with 0.1% Ca was labeled as C1, and the alloy with 0.5% Ca was labeled as C2. To obtain the C1 (0.1% Ca) and C2 (0.5% Ca) alloys from the Mg–XCa–1.5Zn–RE–Zr system, a batch calculation was performed, and high-purity master alloys Mg–15Ca (15% Ca), Mg–20Zn (20% Zn), and a standardized WE43 alloy containing the other elements from the system were used. Cast ingots and samples for experiments are presented in Figure 1.

After casting, the resulting samples were subjected to a heat treatment under a controlled argon atmosphere for homogenization at 420 °C for 12 h, followed by slow cooling.

### 2.2. Microstructural Characterization and Mechanical Properties

Later in the study, after completing the heat treatment, the ingots were sectioned using a Metacut 302, Metkon, Bursa, Turkey cutting machine embedded in resin using Ecopress 52, Metkon, Bursa, Turkey and the surface preparation was performed with a Forcipol 202, Metkon, Bursa, Turkey followed by chemical etching.

- Optical microscopy was conducted using a Leica DMI5000 M, Wetzlar and Mannheim, Germany research microscope.
- EDS and electron microscopy were performed with the Thermo Scientific Quattro C microscope, Brno, Czech Republic.
- strong>∙ For XRD analysis, an X’Pert PRO MPD X-ray, Panalytical, Almelo, The Netherlands, diffractometer was used.
- strong>∙ For spectrometry, two devices were used in parallel for improved accuracy of the chemical composition: the Foundry Master Smart laboratory optical emission spectrometer (Wetzlar and Mannheim, Germany) and the SPECTRO XEPOS energy-dispersive X-ray fluorescence (ED-XRF) spectrometer, (Wetzlar and Mannheim, Germany).
- strong>∙ The UMTR 2M-CTR tribometer, version 1.122.245, was used for surface characterization, and the UMT Test Viewer, CP4–2.16.93+ software was used to assess tribological behavior.
- strong>∙ OriginPro 8.5 was used to process the XRD patterns, and VoltaMaster 4-version 6.0.2.25130 was used to process the electrochemical corrosion tests and surface analysis.

### 2.3. Characterization of Electrochemical Corrosion Resistance

The PGP201 potentiostat (linear and cyclic voltammetry measurements) was used for electrochemical corrosion investigations. The VoltaMaster 4 software was used for experimental data collection and processing. For the potentiodynamic measurements, a three-electrode corrosion cell was used (the glass cell with a working electrode, a Pt wire electrode served as the auxiliary electrode, and a saturated calomel electrode served as the reference). The samples (flat discs with a diameter of 10 mm and a thickness of 1 mm) were mounted in the working electrode using Teflon washers, allowing the creation of flat circular surfaces of up to 0.8 cm^2^. For the samples studied here, the surface exposed to the corrosion environment was S = 0.503 cm^2^. A 3.5% NaCl solution at 25 °C served as the electrolyte, and the solution was continuously aerated using a magnetic stirrer. The working conditions for the measurements were as follows:

- OCP registration.
- Linear anodic polarization: Potential range (−300 to +300) mV relative to the open-circuit potential, with a potential scan rate of dE/dt = 0.1 mV/s.
- Cyclic polarization: Potential range (−500 to +2200) mV, with a potential scan rate of 10 mV/s.

The Vega Tescan LMH II scanning electron microscope (30 kV, SE detector, high pressure) was used to examine the surface structure of both freshly polished and electrochemically corroded samples.

An EDX QUANTAX QX2 detector, made by Bruker, Billerica, MA, USA, attached to an electron microscope, was used to assess the chemical compositions of different surface areas of untreated alloys and the surfaces of corroded samples.

The chemical composition and corrosion resistance determinations were carried out through five and three determinations, respectively, and the paper presented their average values accompanied by the characteristic standard deviations (the StDev calculation was performed in Excel). 

## 3. Results

### 3.1. Microstructural Analysis

#### 3.1.1. Optical Microscopy

**Figure 2:**
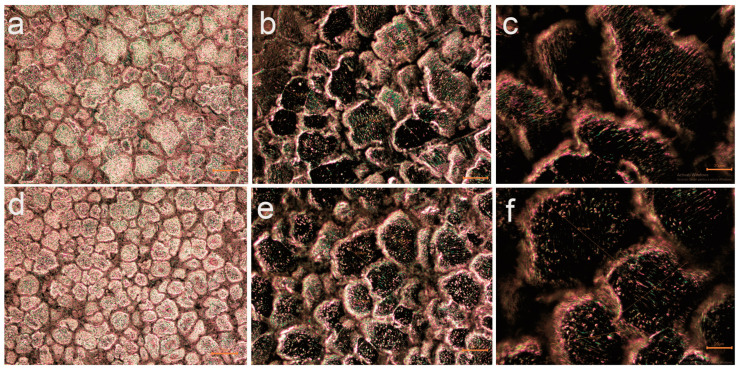
Optical images of the C1 alloy: (**a**) 100×, (**b**) 200×, and (**c**) 500×, and the C2 alloy: (**d**) 100×, (**e**) 200×, and (**f**) 500×.

The general grain structure was evaluated by analyzing the samples at magnifications of 100×, 200×, and 500×. Results are given in Figure 2.

The images do not reveal defects such as segregations, porosities, cracks, or significant voids, highlighting a high-quality alloy manufacturing process. Based on the optical microstructural analysis, a refinement of the grain structure and a reduction in grain size were observed with an increase in Ca content from 0.1% to 0.5% in the alloy composition.

#### 3.1.2. Scanning Electron Microscopy (SEM)

**Figure 3:**
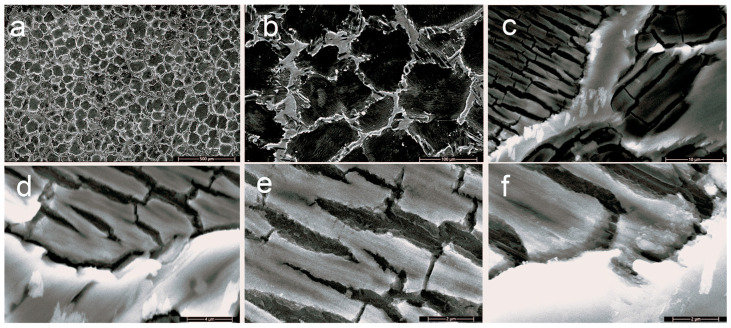
SEM pictures of the C1 alloy at the following magnifications are shown in this figure: (**a**) 100×, (**b**) 500×, (**c**) 1000×, (**d**) 5000×, (**e**) 10,000×, and (**f**) 20,000×.

**Figure 4:**
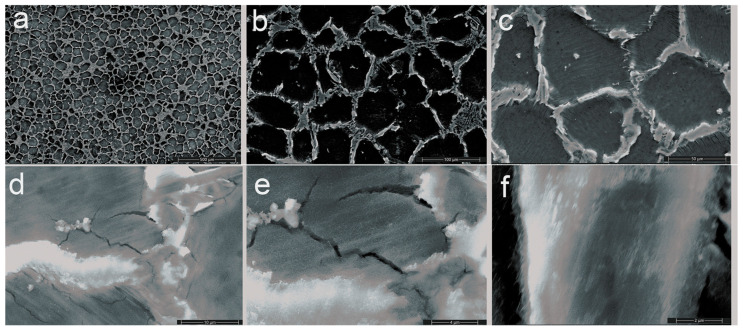
SEM pictures of the C2 alloy at the following magnifications are shown in this figure: (**a**) 100×, (**b**) 500×, (**c**) 1000×, (**d**) 5000×, (**e**) 10,000×, and (**f**) 20,000×.

Scanning electron microscopy (SEM) (ZEISS, Jena, Germany) was used to observe the secondary phases and their distribution, and the selective images are given in Figure 3 and Figure 4.

The Mg–XCa–1.5Zn–RE–Zr alloy system (x = 0.1, 0.5%) highlights the formation of a polyhedral Mg grain structure, while the increase in Ca content and the high percentage of zinc lead to the formation of ternary Ca–Mg–Zn compounds, which exhibit a lamellar structure and are stabilized at the grain boundaries.

### 3.2. Energy-Dispersive X-Ray Spectroscopy (EDS) and X-Ray Difraction (XRD)

**Figure 5:**
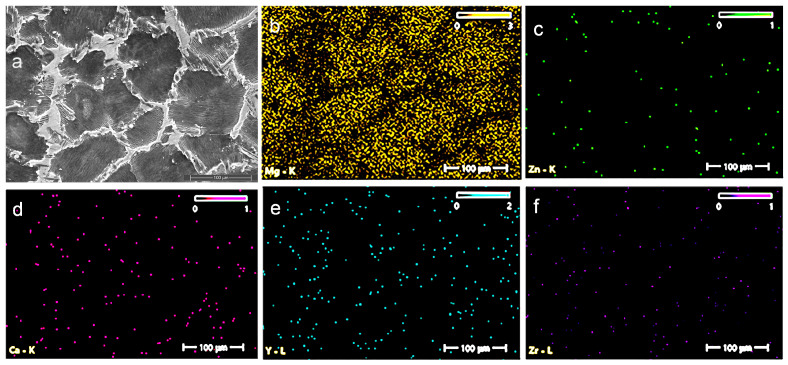
EDS images illustrating the chemical analysis and elemental distribution on the surface of the C1 alloy: (**a**) C1 alloy sample, (**b**) Mg, (**c**) Zn, (**d**) Ca, (**e**) Y, and (**f**) Zr.

**Figure 6:**
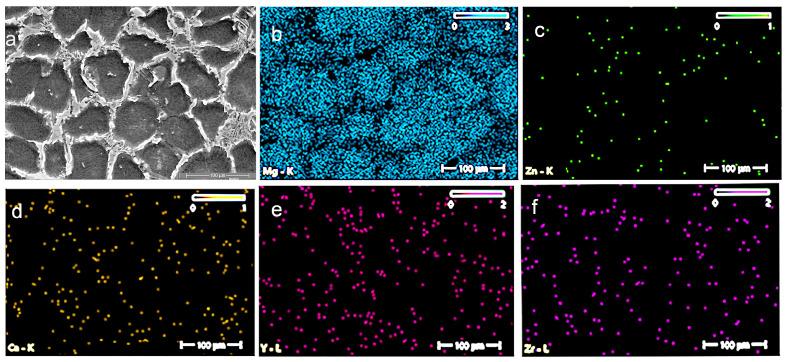
EDS images illustrating the chemical analysis and elemental distribution on the surface of the C2 alloy: (**a**) C2 alloy sample, (**b**) Mg, (**c**) Zn, (**d**) Ca, (**e**) Y, and (**f**) Zr.

Figure 5 and Figure 6 present the main element distributions for the C1 and C2 alloys, respectively. In Table 1, the chemical compositions of C1 and C2 are given (values are average values from three determinations).

Analysis results of the chemical compositions of the alloys studied by spectrometry are given in Table 1.

**Figure 7:**
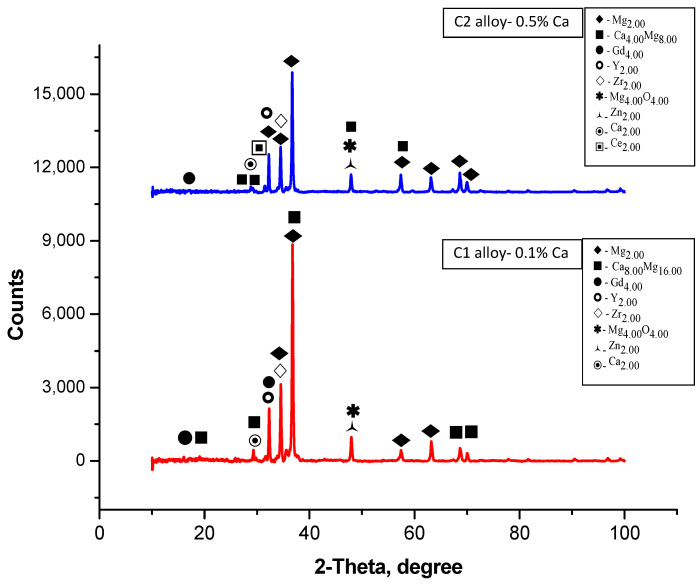
XRD patterns for C1 and C2 samples.

X-ray Diffraction (XRD): XRD analyses were used to identify the intermetallic phases, employing a diffractometer with a Cu–Kα source (λ = 1.5406 Å), and the spectrum is given in Figure 7.

### 3.3. Electrochemical Corrosion Behavior

**Figure 8:**
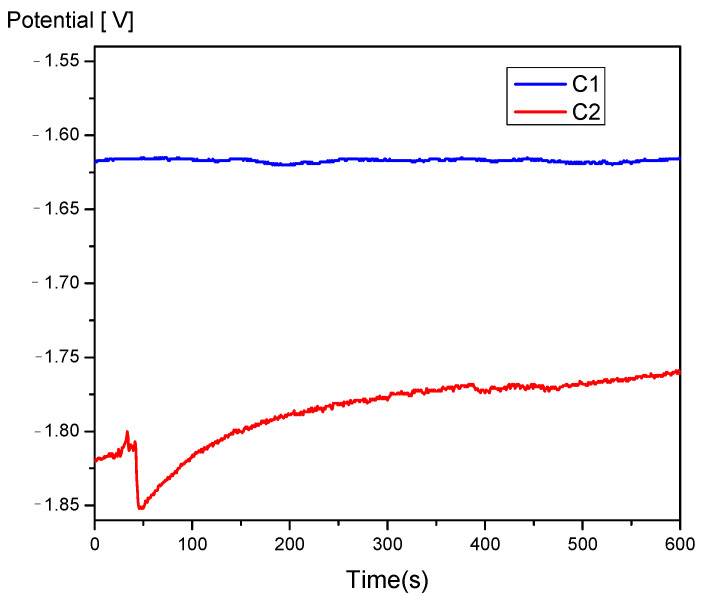
Open-circuit potential in 3.5% NaCl electrolyte.

The corrosion potential (Ecor) represents the thermodynamic probability of a chemical process occurring on the surface of an alloy, meaning it expresses the corrosion tendency of the alloy immersed in an electrolytic medium. It represents the electric potential of the corrodible surface in an electrolyte, measured against a reference electrode, in open-circuit conditions and in the absence of an electric current (at zero current intensity, when the surface electrochemical reaction is at thermodynamic equilibrium). For this reason, it is also referred to as the open-circuit potential (OCP), a term often used when the electrode potential is measured directly with a high-impedance input millivoltmeter [27]. Based on these considerations, the C1 sample, shown in Figure 8, can be considered more resistant to electrochemical corrosion, although the difference between the two samples is not significant.

The results presented in Table 2 are the average of three experiments.

The instantaneous corrosion rate is determined using the polarization resistance method. This method is used to determine the corrosion current at the corrosion potential of the metal or alloy, based on the linear polarization curve obtained for relatively small overpotentials.

**Figure 9:**
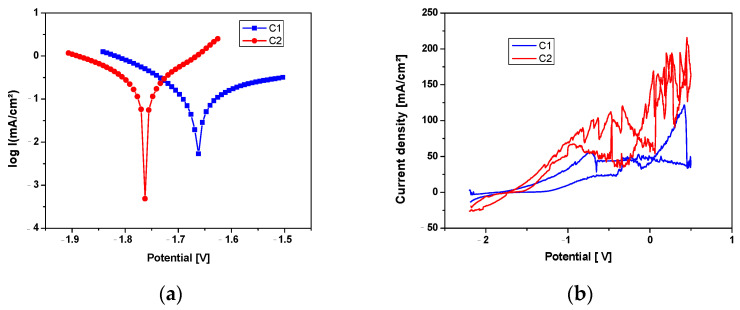
Tafel diagrams in (**a**) and cyclic diagrams in (**b**).

One of the methods for characterizing corrosion processes is cyclic potentiodynamic polarization Figure 9b. To obtain cyclic polarization curves, also called cyclic voltammograms, the polarization of the material is performed continuously at a known potential scan rate (mV/s), and the circuit current is recorded. The potential sweep and current variation readings are carried out automatically, resulting in a continuous curve without discrete data points. 

**Figure 10:**
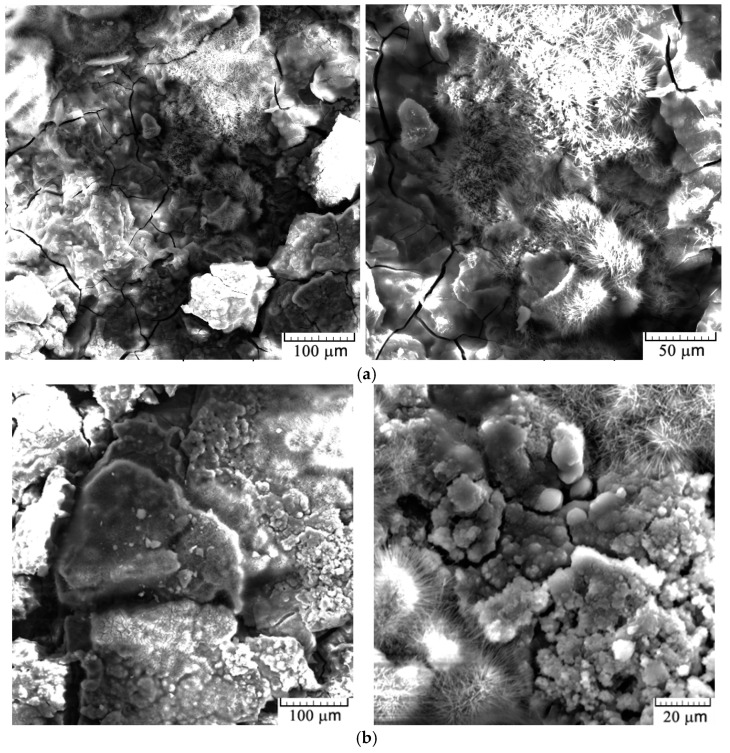
SEM images of the alloy C1 (**a**) and C2 (**b**) after electrochemical corrosion tests.

Figure 10 shows that the surface analysis of the experimental samples, following the corrosion resistance tests, reveals a uniformly corroded surface. 

**Figure 11:**
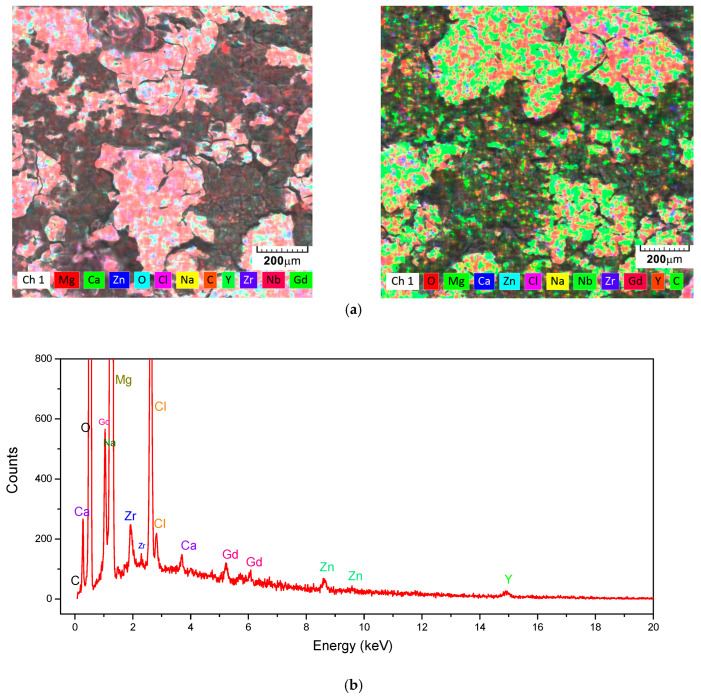
Surface examination following testing for electrochemical corrosion. The primary elements found on the surface for C1 (**left**) and C2 (**right**) are mapped out in (**a**), and the energy spectrum of those elements is shown in (**b**).

**Figure 12:**
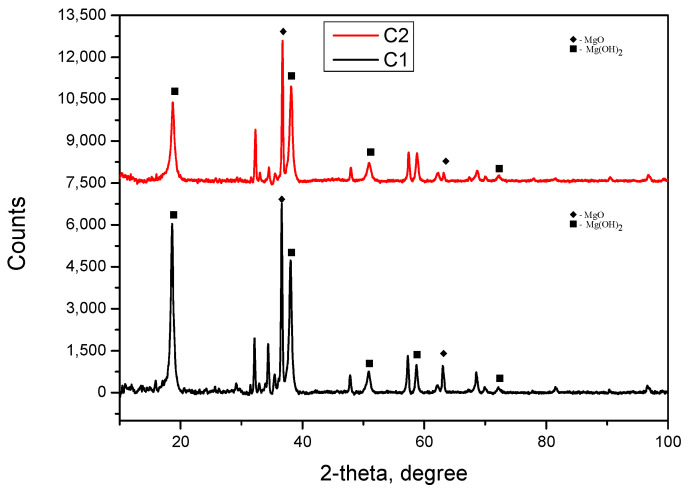
XRD patterns for C1 and C2 samples after polarization test in a 3.5% NaCl solution.

In addition to the base elements of the alloy, shown in Table 1, the elements oxygen, chlorine, and sodium were identified on the surface of the samples after the corrosion test, as shown in Figure 11 and Table 3. These elements appeared as a result of the interaction between the electrolyte solution (saline solution) and the metallic sample. Figure 12 shows the results of the XRD analysis performed on the corroded surface of the material.

On both samples, the formation of an oxide layer was observed, primarily magnesium-based, along with the presence of salt deposits from the saline solution and other compounds based on Cl or Na, as indicated in Table 3.

### 3.4. Mechanical Properties Testing. Determination of the Longitudinal Elastic Modulus Through Micro-Indentation Testing

**Figure 13:**
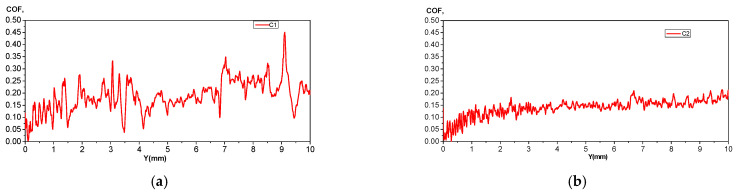
Apparent coefficient of friction (COF) for (**a**) C1 and (**b**) C2 alloys.

The apparent coefficient of friction reflects the degree of surface damage and represents an important variable in analyzing the specific scratch resistance of surfaces. The micro-scratch tests were conducted using a UMTR 2M-CTR tribometer, which employs a sharp stylus to create a controlled, continuous micro-scratch mark on the material’s surface Figure 13. The experiments were conducted with a maximum vertical force of 10 N, moving the table over a 10 mm distance in 60 s at a speed of 0.167 mm/s. An Nvidia cutting blade with a 0.4 mm tip radius was used for the micro-scratch tests.

**Figure 14:**
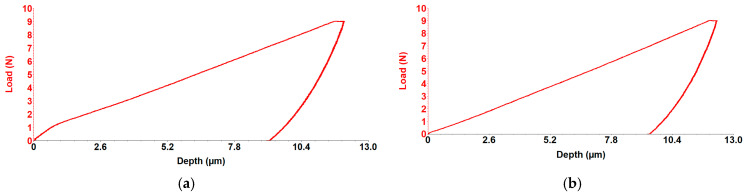
Load-depth curve graph for (**a**) C1 and (**b**) C2 alloys.

For the C1 and C2 alloys, hardness and longitudinal elastic modulus were measured using the micro-indentation method with the UMTR 2M-CTR tribometer. Representative indentation curves are given in Figure 14 and the average values (Stdev) for Stiffness, Depth, Young’s Modulus, and Hardness are given in Table 4. The test specimens were prepared in the form of discs with a diameter of approximately 50 mm and a thickness of about 3 mm. A Rockwell-type diamond penetrator was used, featuring a 120º tip angle and a spherical tip with a radius of 200 μm, applying a maximum force of 10 N.

## 4. Discussion

The results obtained in this study highlight several fundamental aspects regarding the influence of Ca concentration on the microstructure, mechanical properties, and electrochemical corrosion behavior of Mg–Ca–Zn–RE–Zr alloys. The selection of compositions with 0.1% and 0.5% Ca allowed for a detailed investigation of the impact of this element on the alloy characteristics, providing a clear perspective on the advantages and limitations of these materials for orthopedic applications.

The grains exhibit a polyhedral shape with sizes ranging between 50 and 100 µm. At a magnification of 500×, the images clearly reveal the grain boundaries, highlighting a homogeneous microstructure with uniform solidification and no visible segregations.

Dark zones at the grain boundaries suggest the presence of Mg–Ca phases identified by XRD as Ca_8_Mg_1__6_ and Ca_4_Mg_8_.

Uniformly dispersed inclusions are observed, which may represent secondary phases, ensuring a dispersion-strengthening mechanism.

Increasing the Ca concentration from 0.1% to 0.5% led to significant grain refinement, reducing their average size and contributing to the formation of the Mg_2_Ca intermetallic phase. This intermetallic phase serves two purposes: first, it strengthens the matrix by dispersion, increasing the alloy’s mechanical strength; second, it encourages the development of a passive Mg(OH)_2_ protective layer in physiological solutions. Nevertheless, variations in the coefficient of friction and heightened vulnerability to microcracks were noted at greater Ca concentrations (0.5%), most likely as a result of internal pressures brought on by the uneven secondary phase distribution. The addition of zinc to the alloy stabilized the surface structure, which influenced the electrochemical behavior by reducing the corrosion rate and generating a continuous protective layer. Thermal stability and reduced galvanic corrosion propagation may be due to the growth of intermetallic Mg or Zn-based compounds, while zirconium provided additional grain refinement, optimizing the fatigue resistance of the alloys.

For the C1 alloy, the most intense peak is located around the angle of 36.75° (°2Theta), identifying the presence of the intermetallic phase Ca_8_Mg_1__6_, as well as the crystalline phases Mg_2_ and Gd_4_. These may suggest, through correlation and simplification, the presence of the Mg_2_Ca phase, as Mg_2_Ca has an atomic ratio of 2:1 (Mg:Ca), which could be simplified to match the label Ca_8_Mg_1__6_. Other smaller but distinct peaks are distributed across the scale, indicating the presence of other minor crystalline phases.

For the C2 alloy, the most intense peak is located around the angle of 36.67° (°2Theta), identifying the presence of the Mg phase and the intermetallic phase Ca_4_Mg_8_, as well as the crystalline phases Mg_2_ and Gd_4_. These may suggest, through correlation and simplification, the presence of the Mg_2_Ca phase, as Mg_2_Ca has an atomic ratio of 2:1 (Mg:Ca), which could be simplified to match the label Ca_4_Mg_8_.

Mg–Ca–Zn–RE–Zr alloy demonstrates corrosion resistance influenced by calcium concentration, with the C1 alloy exhibiting a lower corrosion rate compared to the C2 alloy. The generalized corrosion behavior and the reduced size of the corrosion compounds favor the degradation process of the implantable element.

The corrosion process parameters, shown in Table 2, indicate better polarization resistance for the C1 sample, resulting in a lower corrosion rate for this alloy. The higher anodic branch value for the C1 sample indicates more pronounced oxidation of this sample, which contributes to an increased level of alloy protection but may also lead to deeper corrosion of the surface layer. The more pronounced oxidation of the C1 sample was also confirmed by chemical analyses performed on the surface after the corrosion process, as shown in Table 3 and Figure 11.

From the Tafel curves in Figure 9a, it can be observed that the samples exhibit similar behavior, with a lower corrosion potential for the C1 sample and a higher anodic branch value for the C1 sample, while the reduction processes on the surface are similar for both experimental samples.

For biodegradable alloys, two processes primarily influence their corrosion resistance: first, the stability of the passivation layer (generally the oxide of the main element, but in this case, a layer composed of multiple oxides) against ion attack from the solution, and second, the alloy’s intrinsic resistance to corrosion. In this case, with an alloy containing intermetallic compounds and the possibility of forming multiple zones with micro-galvanic cells, the alloy’s resistance will rely on the stability of the oxide layer formed on the surface.

By analyzing cyclic polarization curves, information can be obtained about the type of electrochemical process occurring at the electrode/environment interface (such as generalized corrosion, localized corrosion, passivation, reduction, and oxidation of species in the solution), as well as the evaluation of characteristic potentials (corrosion potential, breakdown potential, re-passivation potential, and protection potential).

From the graphs in Figure 9b, it can be observed that the C2 sample undergoes a more pronounced corrosion process, accompanied by gas formation and its release through the destruction of the oxide layer formed due to the interaction between the electrolyte and the experimental alloy. The corrosion occurring on the surface of these alloys is a combination of pitting corrosion, occurring at a relatively high rate and in multiple areas on the surface, which over a short period transforms into generalized corrosion. This is accompanied by the dissolution of the material into the electrolyte solution and re-passivation of the surface through oxidation.

The analysis of the surface of the experimental samples after the corrosion resistance tests shows a generalized corroded surface with a layer of reaction compounds homogeneously distributed over the entire surface, as shown in Figure 10. The corrosion compound layer is interrupted by numerous cracks, pores, or microstructural inhomogeneities.

From the SEM images taken on the corroded surface of the alloy, the formation of oxides is observed, especially magnesium-based oxides (those with a needle-like appearance), as well as other oxides with different structures [28]. The presence of cracks and pores will favorably contribute to the smaller final size of the degradation products, which may cause fewer complications during their elimination or resorption from the biological system [29].

Following surface corrosion, a complex layer of oxides, hydroxides, carbonates, and salts has formed on the surface, which has a protective role in the first part by passivating the surface and which subsequently passes into the electrolyte solution. XRD analysis, with the results presented in Figure 12, performed on the corroded surface of the material, revealed the presence of magnesium oxides and hydroxides, confirming the SEM images, Figure 10, and the element distributions, Figure 11, in which these compounds were structurally identified. In addition to magnesium oxides and hydroxides, other oxides of Zn, Ca, Y, Gd, and Zr are present in different proportions in the layer formed on the surface following the interaction between the experimental alloy and the electrolyte, and confirmation of the representative angles requires further investigations.

The corrosion of alloys in contact with an electrolyte solution is caused by the continuous ion exchange between the solution and the alloy, an interaction generally interrupted by a passivation layer, usually composed of oxides formed on the alloy’s surface. In the case of magnesium-based alloys and other known and studied biodegradable alloys (such as Fe or Zn-based), the passivation layer does not have high resistance over extended periods, as observed with titanium or CoCr-based alloys. This layer breaks down and subsequently reforms after the corroded layer is removed from the surface.

As seen in Figure 9b, the cyclic corrosion diagram appears to fluctuate because of this slow corrosion, which is followed in the case of magnesium alloys by hydrogen release [30]. It is advised that more research be done on the size of the degradation compounds and the quantity of hydrogen emitted by these alloys in electrolyte settings in light of the analysis of the experimental data.

Tribological study, however, showed that 0.1% Ca alloys have greater coefficient of friction fluctuations and are less susceptible to microcrack development, which restricts their use in applications involving cyclic mechanical loads. The predictability of implant longevity suggests that C2 would be preferable for load-bearing applications requiring extended functional stability, while C1 may be more suitable for applications where localized corrosion protection is prioritized, albeit with the necessity of additional surface treatments to enhance wear resistance.

Because of its porous structure, trabecular or spongy bone has a substantially lower elastic modulus, also known as Young’s modulus, than cortical bone, which is much denser. Research indicates that the Young’s modulus of cortical bone is roughly 18 GPa, but that of trabecular bone ranges from 0.7 to 1.7 GPa. Because bone is a biological substance with a particular porosity in trabecular bone, a certain level of hydration, material content, and other factors influenced by age and sex, these variations in the elastic modulus occur [31,32]. Rahman et al. (2024) [33] investigated the as-cast WE43 alloy and determined an elastic modulus of approximately 19 ± 1.27 GPa. By incorporating Ca and Zn into the alloy, they developed an enhanced material with an elastic modulus of 18.6 ± 8.0 GPa, comparable to WE43, but exhibiting superior mechanical properties, including higher tensile strength and improved ductility. The experimentally obtained values of 14.8 GPa (C1 alloy) and 18.6 GPa (C2 alloy) recommend, from a biomechanical perspective and for avoiding the stress-shielding effect, the use of both alloys as biodegradable implants.

The depth and width of the scratches are larger and steeper for the C1 alloy (Figure 13a, demonstrating superior elasticity, which provides better absorption of mechanical shocks. The C2 alloy exhibits smaller COF fluctuations, Figure 13b, with shallower scratches (11.07 µm), an average hardness value of 0.67 GPa, and a Young’s modulus of 18.6 GPa, indicating higher abrasion resistance and lower localized wear. This can be attributed to the formation of hard phases, which cause localized increases in friction and a transition between different wear modes.

The C2 alloy has a slightly higher elastic modulus, indicating increased stiffness and higher susceptibility to cracks and fractures.

The alloy with 0.1% Ca exhibits a slightly higher apparent coefficient of friction (COF), 0.19 (C1) > 0.14 (C2), which shows a greater scratch resistance for C2 alloy. Comparing the average stiffness values 2.52 N/µ (C2) > 2.2 N/µ (C1) and depth values 13.35 µm (C1) > 11.07 µm, (C2) indicates a more stable tribological behavior for the C2 alloy. An average hardness of 0.67 GPa (C2) compared to 0.57 GPa (C1), and a Young’s modulus of 18.6 GPa (C2) compared to 14.85 GPa (C1), characterize the C2 alloy by a uniform distribution of mechanical strength, suggesting a more balanced interaction between the contacting surfaces and a more controlled wear behavior, Figure 14.

The variable Ca concentrations (0.1% and 0.5%) demonstrated a direct influence on the microstructure of the Mg–Ca–Zn–RE–Zr alloys. At 0.5% Ca, grain refinement and intermetallic phase formation resulted in increased hardness and abrasion resistance with less fluctuating tribological behavior. The C2 alloy may be more suitable for abrasion resistance or mechanical applications requiring long-term stability, while the alloy with C1 can be employed in applications where friction coefficient and corrosion resistance behavior are prioritized, although its use is limited in environments subjected to high cyclic stresses.

According to the specialized literature, the addition of Ca in lower concentration ranges (0.4–1 wt.%) facilitates grain refinement, thereby enhancing the mechanical properties and structural stability of the material. This modification also improves the alloy’s corrosion resistance by promoting the formation of a protective Mg(OH)_2_ layer. However, an excessive increase in Ca content (>4 wt.%) can lead to a less homogeneous microstructure and embrittlement due to the excessive formation of the intermetallic Mg_2_Ca phase, which accelerates galvanic corrosion and subsequently reduces the implant’s service life. Additionally, high Ca concentrations contribute to a significant decrease in ductility, compromising the alloy’s ability to withstand mechanical loads in orthopedic applications [34].

The study demonstrates the influence of Ca concentration on the microstructure, mechanical, and tribological properties of Mg–Ca–Zn–RE–Zr alloys, highlighting their potential for orthopedic applications. However, for clinical validation, further research is required to optimize degradation through advanced corrosion protection strategies, enhance the strength-ductility balance via severe plastic deformation techniques, and assess biocompatibility through preclinical studies. Additionally, comparative analyses with conventional titanium-based implants or other biodegradable Mg-based alloys are essential to establish their long-term viability and clinical applicability.

## 5. Conclusions

The findings underscore the critical importance of precise control over composition and microstructural parameters in the design of advanced Mg-based alloys, engineered to enhance both mechanical performance and biocompatibility within complex physiological environments. Analyzing the experimental results, a few conclusions can be drawn:

Increasing Ca content will highlight the grain refinement and reduce the average size.

In addition to the Mg-based phase (solid solution), different intermetallic compounds are formed in the alloys.

Both alloys present similar corrosion behavior with a slightly higher Rp for C1. An thick layers form on both cases.

The variation in Ca concentration between 0.1% and 0.5% showed a significant correlation with key mechanical properties such as elastic modulus, hardness, and strength, all of which are essential for structural and functional compatibility with human bone. Both alloys exhibited experimentally determined elastic modulus values, 14.85 GPa for C1 and 18.60 GPa for C2, within the range of human bone (10–30 GPa). When selecting these alloys for biomaterial applications, the specific characteristics of each should be taken into account: the C2 alloy is ideal for applications requiring high mechanical strength and long-term stability, whereas the C1 alloy is more appropriate when localized corrosion resistance is prioritized, provided additional surface treatments are employed to enhance its wear resistance.

However, for clinical translation, further investigations are required to refine degradation kinetics through advanced corrosion protection strategies, optimize the strength-ductility balance via severe plastic deformation techniques, and assess biocompatibility through comprehensive preclinical evaluations.

## Figures and Tables

**Table 1:** Chemical composition of the alloy (C1) and the alloy (C2).

System	Mg	Ca	Zn	Y	Zr	Nd	Dy	Gd
C1 (spark)	94.7	0.16	1.42	1.19	0.6	0.46	0.4	0.17
C1 (XRF)	92.68	0.13	3.4	-	0.38	0.1	-	-
C2 (spark)	92	0.39	2.78	2.10	0.6	0.69	0.4	0.28
C2 (xrf)	90.81	0.42	3.79	-	1.3	0.1	-	-

**Table 2:** Corrosion Process Parameters (E: corrosion potential, i_corr_: corrosion current, Rp: polarization resistance, V_corr_: corrosion rate, βc: the cathodic slope of the Tafel diagram, and βa: the anodic slope of the Tafel diagram).

System	Corrosion Process Parameters					
	E(I = 0) (mV)	i_corr_ (mA/cm^2^)	Rp (ohm/cm^2^)	v_corr_ (mm/year)	β_c_ (mV/dec)	β_a_ (mV/dec)
C1	−1662.7	0.14	327.4	3.39	−185.0	461.6
C2	−1762.5	0.16	122.3	3.79	−146.1	122.8

**Table 3:** Chemical composition of the C1 and C2 samples after the electrochemical corrosion tests (1 mm^2^ area).

Sample/ Chemical Elements	O%		Mg%		Cl%		Na%		Y%		Zn%		Gd%		Ca%		Zr%		C%	
	wt	at	wt	at	wt	at	wt	at	wt	at	wt	at	wt	at	wt	at	wt	at	wt	at
C1(0.1%)	57.3	64.3	30.2	22.3	2.3	1.2	1.6	1.3	0.9	0.2	0.25	0.07	0.11	0.01	0.1	0.05	0.1	0.02	7.1	10.6
C2(0.5%)	56.7	66.6	29.9	22.4	3.2	1.7	0.9	0.7	1.3	0.3	0.3	0.1	0.4	0.1	0.5	0.2	0.30	0.1	6.6	10
EDS error	5		2		0.2		0.2		0.1		0.1		0.1		0.1		0.1		10	

Mention: carbon element is present based on SEM–EDS system chamber contamination and should not be taken in consideration; St Dev: 0 ± 3; Mg: ±1; Cl: ±0.1; Na: ±0.1; Y: ±0.1; Zn: ±0.05; Gd: ±0.09; Ca: ±0.1; and Zr: ±0.1.

**Table 4:** Average Values (Stdev)for Stiffness, Depth, Young’s Modulus, and Hardness for Alloys C1 and C2.

	Avg. C1 Alloy	Avg. C2 Alloy
Stiffness (N/µm)	2.2 (±0.1)	2.52 (±0.1)
Depth (µm)	13.35 (±0.6)	11.07 (±1.1)
Young’s (Gpa)	14.85 (±1.1)	18.60 (±1.7)
Hardness (Gpa)	0.57 (±0.02)	0.67 (±0.06)

## Data Availability

The original contributions presented in the study are included in the article, further inquiries can be directed to the corresponding authors.
